# The Anterior Eye Chamber as a Visible Medium for In Vivo Tumorigenicity Tests

**DOI:** 10.1093/stcltm/szac036

**Published:** 2022-06-06

**Authors:** Emi Inagaki, Eri Arai, Shin Hatou, Tomoko Sayano, Hiroko Taniguchi, Kazuno Negishi, Yae Kanai, Yasunori Sato, Hideyuki Okano, Kazuo Tsubota, Shigeto Shimmura

**Affiliations:** Department of Physiology, Keio University School of Medicine, Tokyo, Japan; Japanese Society for the Promotion of Science (JSPS), Tokyo, Japan; Department of Ophthalmology, Keio University School of Medicine, Tokyo, Japan; Department of Pathology, Keio University School of Medicine, Tokyo, Japan; Department of Ophthalmology, Keio University School of Medicine, Tokyo, Japan; Cellusion Inc., Tokyo, Japan; Department of Ophthalmology, Keio University School of Medicine, Tokyo, Japan; Cellusion Inc., Tokyo, Japan; Department of Ophthalmology, Keio University School of Medicine, Tokyo, Japan; Department of Ophthalmology, Keio University School of Medicine, Tokyo, Japan; Department of Pathology, Keio University School of Medicine, Tokyo, Japan; Department of Preventive Medicine and Public Health, Keio University School of Medicine, Tokyo, Japan; Department of Physiology, Keio University School of Medicine, Tokyo, Japan; Department of Ophthalmology, Keio University School of Medicine, Tokyo, Japan; Department of Ophthalmology, Keio University School of Medicine, Tokyo, Japan

**Keywords:** induced pluripotent stem cells, regenerative medicine, teratoma formation assay, anterior chamber

## Abstract

Pluripotent stem cell (PSC)-based cell therapies have increased steadily over the past few years, and assessing the risk of tumor formation is a high priority for clinical studies. Current in vivo tumorigenesis studies require several months and depend strongly on the site of grafting. In this study, we report that the anterior eye chamber is preferable to the subcutaneous space for in vivo tumorigenesis studies for several reasons. First, cells can easily be transplanted into the anterior chamber and monitored in real-time without sacrificing the animals due to the transparency of the cornea. Second, tumor formation is faster than with the conventional subcutaneous method. The median tumor formation time in the subcutaneous area was 18.50 weeks (95% CI 10.20-26.29), vs. 4.0 weeks (95% CI 3.34-.67) in the anterior chamber (*P* = .0089). When hiPSCs were spiked with fibroblasts, the log_10_TPD50 was 3.26, compared with 4.99 when hiPSCs were transplanted without fibroblasts. There was more than a 40-fold difference in the log_10_TPD50 values with fibroblasts. Furthermore, the log_10_TPD50 for HeLa cells was 1.45 and 100% of animals formed tumors at a concentration greater than 0.1%, indicating that the anterior chamber tumorigenesis assays can be applied for cancer cell lines as well. Thus, our method has the potential to become a powerful tool in all areas of tumorigenesis studies and cancer research.

## Graphical Abstract

**Figure:**
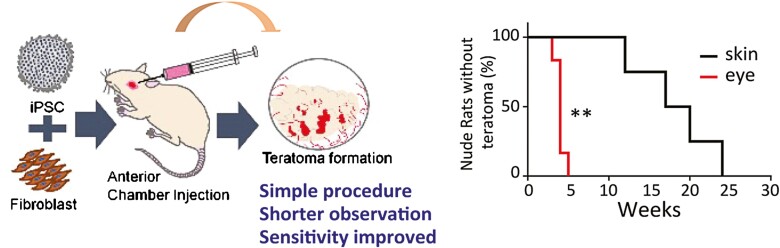

**Significance Statement**

Confirming the safety of cellular products is critical for developing cell therapies. However, current assays to evaluate cell pluripotency and tumorigenic potential require several months. Transplantation in the anterior eye chamber of nude rats is easy, can be monitored in real-time due to corneal transparency, allows for fast teratoma formation, and is highly sensitive to spiking with human dermal fibroblasts.

## Introduction

When human embryonic stem cells (hESCs) were first proposed as a possible source of cells for therapeutic use, the primary concern was the possible tumor formation when transplanted in vivo.^1^ The pluripotent nature of these cells is conventionally shown by the formation of teratomas when grafted into immune-deficient mice.^2^ Studies have shown that the environment in which hESCs are transplanted influences the ability of grafted cells to differentiate.^3^ Subcutaneous transplantation was then shown to be the most permissive for teratoma formation, and subsequent studies have used the subcutaneous location as a gold standard.^4,5^ Hentze et al. also compared various locations that support the formation of teratomas, including the kidney capsule, muscle, subcutaneous space, peritoneal cavity, testis, liver, epididymal fat pad, and found that intramuscular injection of hESCs into severe combined immune deficient (SCID)) mice were the most convenient.^6^

With the development of induced pluripotent stem cells (iPSCs), an additional challenge to test for de novo tumorigenesis, or tumor formation due to transformation, was introduced into the field.^7-9^ Cell therapy involving iPSCs now requires proof showing the lack of residual pluripotent cells and the assurance that transplanted cells do not form tumors. For in vivo analysis, immunodeficient mice such as SCID, nonobese diabetic/severe combined immunodeficient (NOD-SCID), and NOD-*scid IL-2Rγ*^*null*^ (NOG) strains are often used in a subcutaneous transplantation model using Matrigel as a matrix to facilitate cell proliferation.^10^ Most in vivo tumorigenesis studies use an established tumor cell line such as HeLa cells as a positive control. There are arguments that in vitro assays, such as the soft agar colony formation (SACF) assay, are more sensitive than in vivo assays. A previous study reported that image-based screening system for the SACF assay using a high-content cell analyzer (digital SACF assay) saves time and is more sensitive than in vivo tumorigenicity assays.^11^ However, de novo tumor formation is affected by surrounding environmental cues, indicating that in vivo studies must take this influence into consideration.^10,12-15^

Based on the results of our study, we propose that the anterior chamber of the eye is preferable to the subcutaneous space for tumorigenesis studies for several reasons. First and foremost, since the cornea is transparent, cells transplanted into the anterior chamber can be observed in real-time without sacrificing the animals. For this reason, the anterior chamber of the eye has been used to study glomeruli,^16^ and pancreatic islet cells.^17-21^ The anterior chamber will also allow the detection of small clusters of cells along with the formation of feeder vessels, and innervation since the anterior chamber and the cornea are both avascular in normal mice and rats.^22,23^ Here, we show the sensitivity and time course of tumor formation in a new anterior chamber tumorigenesis model.

## Materials and Methods

### Animals

Four-week-old female nude rats (F344/NJcl-rnu/rnu; CLEA Japan, Inc., Tokyo, Japan) were used as immune-deficient recipient animals.^24^ Due to the control of the single recessive gene rnu, homozygotes (mu/mu) are anatomically athymic and immunologically lack T cell function. All animals were handled in full accordance with the Association for Research in Vision and Ophthalmology (ARVO) Statement for the Use of Animals in Ophthalmic and Vision Research. This study was approved by the Animal Research Committee of the School of Medicine of Keio University (Approval Number:15084). Six rats were used for each experiment (3 males and 3 females). The age of rats ranged from 4 to 6 weeks. In consideration of animal welfare, experiments were performed in one eye only. The animals were kept under pathogen-free conditions on a 12-h day/night cycle and were free to access water and food. No animals developed behavioral abnormalities or any general symptoms during the follow-up period.

### Cell Culture

Research grade human induced Pluripotent Stem Cell (hiPSC) lines 201B7,^25^ Ff-MH09s01, Ff-I01s01, and Ff-I01s04 were obtained from the Center for iPS Cell Research and Application Foundation, Kyoto University (CiRA). hiPSCs were expanded under serum-free and feeder-free conditions. Frozen stocks were thawed and seeded at a concentration of 2 × 10^5^ cells/well with Stemfit® AK03N culture medium (Ajinomoto Healthy Supply, Tokyo, Japan) supplemented with 10 µM ROCK inhibitor Y27632 (Nacalai Tesque, Kyoto, Japan) in 6-well culture plates (Sumitomo Bakelite, Tokyo, Japan) coated with 0.6 µg/cm^2^ laminin-511 E8 fragment (iMatrix-511, Nippi, Tokyo, Japan) at 37 °C, 5% CO_2_. The following day, the culture medium was replaced with AK03N without Y27632 and then changed every weekday. iPSCs were passaged with TrypLE Select (Thermo-Fischer Scientific, Waltham, MA, USA) every week on 6-well culture plates coated with iMatrix-511.

Normal adult human dermal fibroblasts (NHDF-c, adult) were purchased from PromoCell (Heidelberg, Germany), cultured in DMEM (Nacalai Tesque) supplemented with 10% fetal bovine serum (Thermo-Fischer Scientific) and 1 mg/mL of penicillin/streptomycin (Nacalai Tesque) under a humidified atmosphere of 5% CO_2_ at 37 °C. The medium was changed every 2-3 days. The initial culture was carried out in 25 cm^2^ tissue culture flasks (Nacalai Tesque) and cells were replated in 75 cm^2^ tissue culture flasks (Nacalai Tesque) for subsequent experiments. HeLa cells (RCB 0007) were purchased from Riken BRC (Riken BioResource Center) and cultured in MEM (Nacalai Tesque) supplemented with 10% fetal bovine serum (Thermo-Fischer Scientific) and 1 mg/mL of penicillin/streptomycin under a humidified atmosphere of 5% CO_2_ at 37 °C. The medium was changed every 2-3 days. HeLa cells reached semiconfluence in 1 week. The initial culture was carried out in 25 cm^2^ tissue culture flasks and was replated in 75 cm^2^ tissue culture flasks for subsequent experiments.

### Subcutaneous Transplantation

A total of 1 × 10^6^ hiPSCs in Matrigel™ (BD Bioscience, 10 mg/mL) were injected subcutaneously in 4-week-old female nude rats (F344/NJcl-rnu/rnu; CLEA Japan, Inc.). Cells were suspended in 5 µL of PBS, and an equal volume of Matrigel was added to make a 10 µL cell suspension for transplantation. Cells were injected using a 1 mL insulin syringe (Terumo Corporation) with a 29G needle. The animals were monitored for 32 weeks. The negative control group was injected with PBS and Matrigel. Animals were observed every day for clinical signs. Tumor formation was evaluated every week. The endpoint of the subcutaneous injection model was when a palpable tumor was detected under the skin. At the end of the experiments, the rats were sacrificed and the teratomas were removed and fixed with 4% paraformaldehyde (PFA) for 24 h. Paraffin sections were stained with H&E for histological observation. Rats were anesthetized with isoflurane (Abbott Scandinavia) or a mix of ketamine (80-100 mg/kg; Fort Dodge) and xylazine (5-10 mg/kg; Phoenix Pharmaceutical).

### Anterior Chamber Transplantation

**Figure 1.**
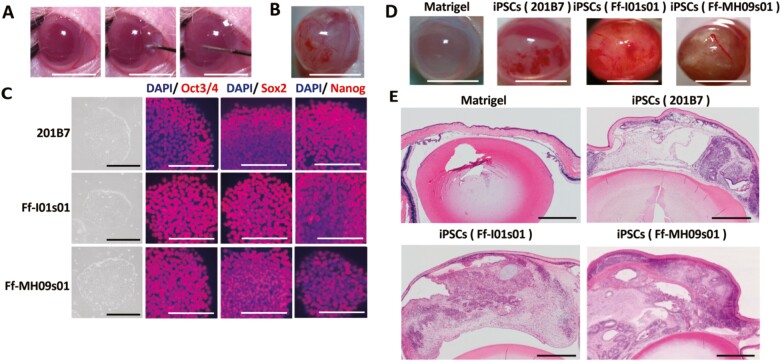
Teratoma formation in the anterior chamber of nude rats. (**A**) Photographs of the anterior chamber of nude rats. Cells were injected into the anterior chamber in three steps by grasping the cornea, puncture, and injection of cells. Rats were monitored weekly for the appearance of tumors and the progression of tumor size. The endpoint of the experiment was when the tumor occupied mostly the whole of the anterior chamber or if noticeable exophthalmos appeared. Scale bars = 5 mm. (**B**) Teratoma formation in the anterior chamber. Transplanted iPSC (201B7, 1 × 10^6^) with Matrigel differentiated into a teratoma that occupied the entire anterior chamber macroscopically after four weeks. Scale bars = 5 mm. (**C**) Characterization of iPSCs. Representative immunohistochemical analysis of iPSCs showed that each iPSC line was positive for OCT3/4, SOX2, and NANOG. The nuclei were labeled with DAPI. Scale bars = 50 μm (in vitro morphology), 50 μm (merge, OCT3/4, SOX2, and NANOG panel). (**D**) Macroscopic images of eyes of nude rats after iPSC transplantation in the anterior chamber. From left to right: Matrigel transplant group, iPSC transplanted group (201B7) (Ff-I01s01) (Ff-MH09s01). Solid tumor filled in the anterior chamber can be recognized without sampling. Scale bars = 5 mm. (**E**) HE staining of cross-sectional images of the eyes of nude rats after iPSC transplantation in the anterior chamber. Histological analysis revealed that teratomas developed in all eyes transplanted with iPSCs (201B7, Ff-I01s01, and Ff-MH09s01), but not in the eyes injected with Matrigel alone, as a negative control. Scale bars = 1 mm.

Cells were injected into the anterior chamber of the eye using a 1 mL insulin syringe (Terumo Corporation) with a 29 G needle in three steps: The nasal side of the eyelid was held with a tweezer applying mild pressure (Fig. 1A), then the eye was punctured with the insulin syringe, bevel down from the limbal side. Finally, cells in Matrigel were injected into the anterior chamber above the iris. Care was taken not to damage the iris to avoid bleeding. All rats recovered from anesthesia with no signs of stress or irritation in the manipulated eye.

### Immunohistochemistry

Immunocytochemical analyses of in vitro samples were performed as described previously.^26^ Briefly, samples were fixed at room temperature for 10 min in 4% formaldehyde in PBS. After 3 washes with PBS, cells were incubated for 15 minutes in Morphosave (Ventana Medical Systems, Inc., Tucson, Arizona). After 2 additional washes with PBS, the samples were incubated for 30 min in 10% normal donkey serum to avoid nonspecific binding. This was followed by overnight incubation at 4 °C with 1:200-diluted mouse anti-Oct 3/4 goat anti-antibody (Santa Cruz Biotechnology, Inc. sc-8628, Dallas, Texas) or 1:200-diluted rabbit anti-SOX2 antibody (Abcam, Cambridge, UK) or 1:200-diluted rabbit anti-NANOG antibody (REPROCELL, Yokohama, Japan) and then washed 3 times in PBS. The samples were then incubated for 2 h in a 1:200 dilution of Cy3-conjugated donkey anti-mouse IgG antibody (Jackson Immuno Research Laboratories, West Grove, PA, final concentration 30 mg/mL) or Cy3-conjugated donkey anti-rabbit IgG antibody (Jackson Immuno Research Laboratories) and washed 3 times in the dark. Finally, samples were mounted on slides with an anti-fading mounting medium containing 4ʹ,6-diamidino-2-phenylindole ([DAPI] 1 mg/mL, Dojindo Laboratories, Kumamoto, Japan). Fluorescent images which were obtained by fluorescent microscopy (Axio Imager; Carl Zeiss, Inc., Weimar, Germany).

Enucleated eyes and skin samples were excised and fixed in 4% paraformaldehyde for at least 18 h. Samples were embedded in paraffin and cut serially into 5 µm sections. Subsequently, the sections were stained with HE staining and subjected to histological analysis by a certified pathologist. Immunohistochemistry (IHC) staining was performed automatically by BOND-MAX™ (IHC staining system, Leica Microsystems GmbH, Leica, Wetzlar, Germany, https://leica-camera.com/). The following antibodies were used in this study: anti-NFP antibody (DAKO, M0762, Agilent, Santa Clara, CA), anti-αSMA (DAKO, M0851), anti-AFP (DAKO, A008), anti-GFAP (DAKO, M0761), anti-Desmin (DAKO, M0760), anti-Ku80 (Cell Signaling Technology, Danvers, Massachusetts), and Toluidine Blue (Merck Millipore, Burlington, Massachusetts). Immunohistochemistry images of rat eyes were obtained with BZ-9000™ microscope (Keyence, Osaka City, Osaka, Japan).

## Results

### Tumorigenicity in the Anterior Chamber of the Eye

First, we established a protocol to inject cells into the anterior chamber of athymic nude rats (F344/NJcl-rnu/rnu). Fig. 1A shows the experimental procedure in a series of photographs. Briefly, the head was fixed and the injection of cells in Matrigel was performed from the limbus. The 29G insulin needle punctured the anterior chamber above the lens, avoiding injury to surrounding tissue such as the cornea, lens, and iris. Finally, 1 × 10^6^ hiPSCs were gently injected with Matrigel (Fig. 1A). Transplanted hiPSCs were the 201B7, Ff-I01s01, and Ff-MH09s01 clones. All 3 cell lines expressed the pluripotency markers OCT3/4, SOX2, and NANOG (Fig. 1C). The transplanted animals were monitored weekly for tumor appearance and progression of tumor size. The endpoint of the anterior chamber model was when the tumors macroscopically occupied the entire volume of the anterior chamber and if the eye showed notable protrusion compared to the fellow eye. After 4 weeks, a solid tumor was observed in the anterior chamber of the eye due to the transparent nature of the cornea (Fig. 1B). Nude rats were sacrificed, and eyes were enucleated. Micrographs of each iPS line are shown in Fig. 1D (left panel). HE sections of enucleated eyes transplanted with each iPSC line (201B7, Ff-I01s01, and Ff-MH09s01) were evaluated. Tumors derived from each iPSC line were definite teratomas composed of multiple tissues originating from the three germ layers (ectoderm, mesoderm, and endoderm) (Fig. 1D, right panels).

### Teratoma Formation

**Figure 2.**
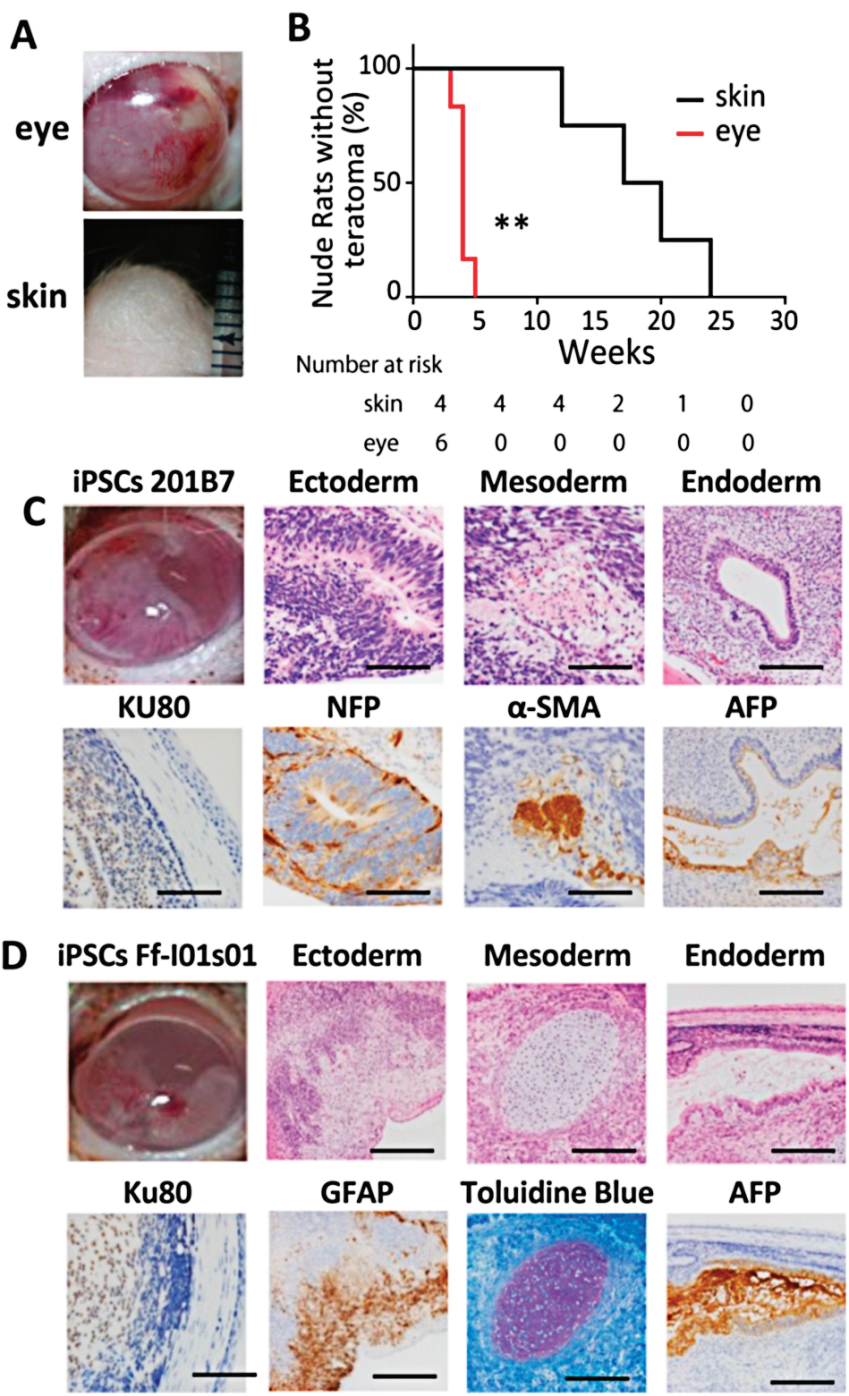
Comparison of the anterior chamber and subcutaneous teratoma formation. (**A**) Macroscopic images of teratoma formed in the skin (injected in subcutaneous tissue, bottom) and in the eye (injected in the anterior chamber, top) (201B7, 1 × 10-6, with Matrigel). (**B**) Kaplan-Meier curve for tumor formation in nude rats injected with iPSCs (201B7) and Matrigel in the anterior chamber and subcutaneous tissue. The median tumor formation period in the subcutaneous area was 18.50 weeks (95%CI 10.20-26.29). (*n* = 4), vs. 4.0 (95%CI 3.34-4.67) in the anterior chamber (*n* = 6). Log-rank test (*P* = .0089). (**C**) Representative images of teratomas formed in the anterior chamber derived from 201B7 iPSC. The presence of three primary germ layers was confirmed by histology and immunohistochemistry. Cells derived from the human origin are demonstrated by positive staining of Ku80. Scale bars = 50 μm. (**D**) Representative images of teratomas formed in the anterior chamber derived from Ff-s01s01 iPSCs. As in 201B7 iPSC, the presence of three primary germ layer derivatives was confirmed by histology and immunohistochemistry. Scale bars = 50 μm.

To compare intraocular and subcutaneous teratoma formation, 1 × 10^6^ 201B7 iPSCs were transplanted into the anterior chamber of the eye or the subcutaneous area of the back skin in two groups of rats. Rats were monitored every week until teratoma formed at each transplant site. After 2-4 weeks, teratomas grew successfully in the anterior chamber. In contrast, approximately 6 months were required to confirm teratomas in subcutaneously injected rats (Fig. 2A). Tumorigenesis was faster in the anterior chamber compared to subcutaneous transplantation. Kaplan-Meier curves revealed that the anterior chamber formed observable tumors much faster than in the skin and that there was less variability in the anterior chamber (Fig. 2B). We injected two iPS cell lines (201B7 and Ff-I01s01s) with Matrigel, and both lines successfully formed teratomas (Fig. 2C, 2D). The tumors arising within anterior chambers were histologically confirmed to be teratomas. Anti-NFP and anti-GFAP staining demonstrated the presence of neuronal lineage cells and pointed to differentiation from the ectoderm, respectively. Anti- αSMA and toluidine blue staining supported smooth muscle and chondrocyte differentiation from the mesoderm, respectively. Anti-AFP staining showed an immature digestive organ differentiated from the yolk sac, a part of the endoderm (Fig. 2C, 2D). Most of the teratoma cells were of human origin, as shown by positive staining with the anti-human nucleus antibody (Ku80). Some fibroblasts and vascular tissue surrounding the teratoma were negative for Ku80, suggesting that they were a reactive change derived from the recipient rat.

### Sensitivity of the Anterior Chamber Tumorigenesis Assay

**Figure 3.**
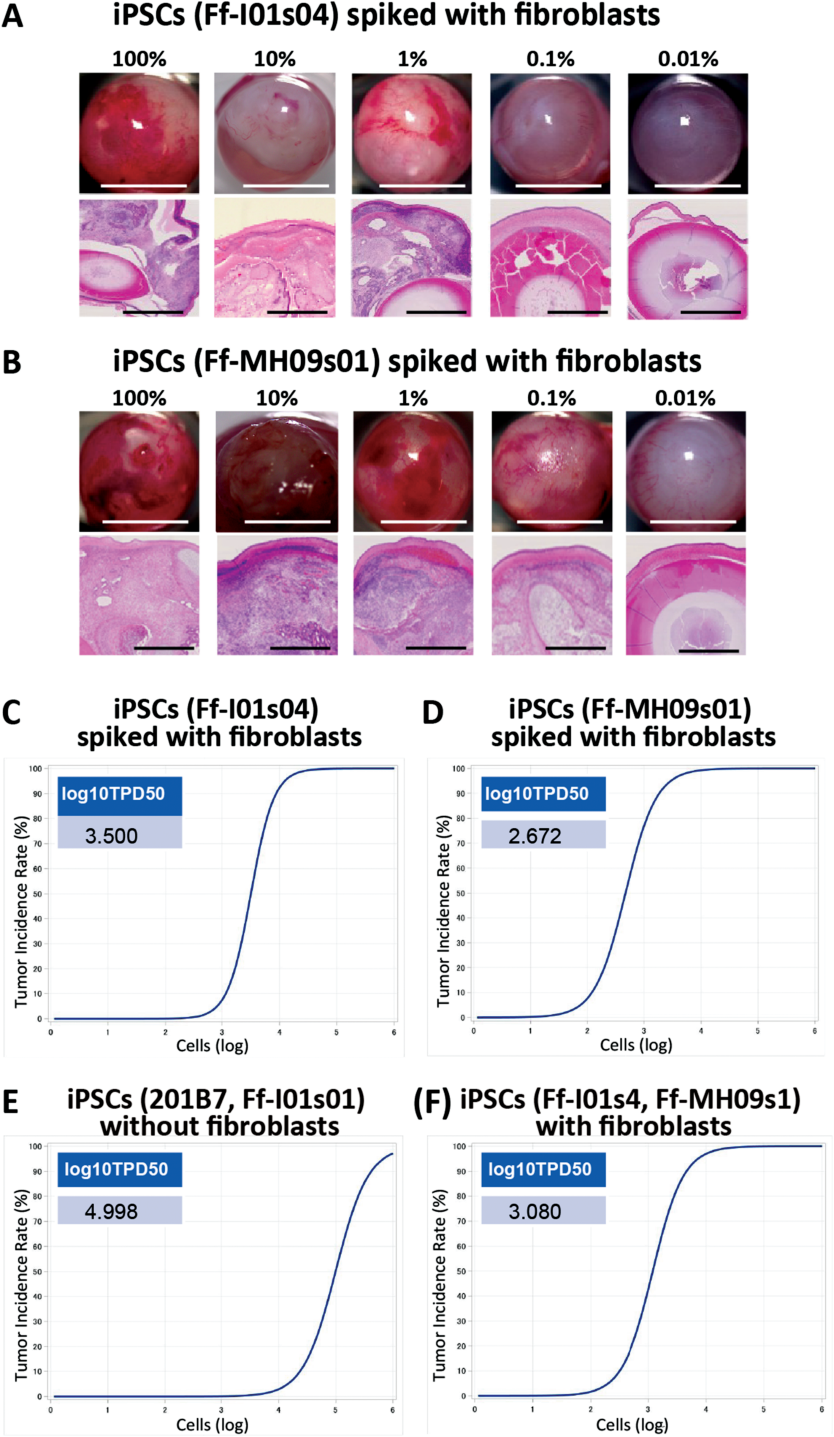
Spike tests of iPSCs with human dermal fibroblasts. (**A**) Images of nude rat eyes transplanted with various concentrations of Ff-01s04 iPSCs (upper panel, Scale bars = 5 mm). HE-stained section of the eyeball (lower panel). [*n* = 6 (male *n* = 3, female *n* = 3)]. Teratomas were not formed with concentrations lower than 0.1%. Scale bars = 1 mm. (**B**) Images of nude rat eyes transplanted with various concentrations of Ff-MH09s01 iPSCs (upper panel). HE-stained section of the eyeball (lower panel) [*n* = 6 (male *n* = 3, female *n* = 3)]. Teratomas were not formed with concentrations lower than 0.01%. Scale bars = 500 μm. (**C**) Line graph showing the minimum dose that can generate a tumor in 50% of animals transplanted with Ff-01s04 iPSC spiked with fibroblasts. log_10_TPD50 was 3.500. (**D**) Line graph showing the TPD50 for Ff-MH09s01 iPSC spiked with fibroblasts. log_10_TPD50 was 2.672. (**E**) Line graph showing the minimum dose that can generate a tumor in 50% of animals transplanted with 201B7 or Ff-01s01 iPSCs without fibroblasts. log_10_TPD50 was 4.998. (**F**) Line graph showing the minimum dose that can generate a tumor in 50% of animals transplanted with Ff-01s04 or Ff-MH09s01 iPSCs spiked with fibroblasts. Log_10_TPD50 was 3.500.

After we demonstrated that our assay could efficiently promote the formation of teratomas from a relatively large number of hiPSCs, we sought to evaluate the sensitivity of the assay. We injected different numbers of cells (1 × 10^2^, 1 × 10^3^, 1 × 10^4^, 1 × 10^5^, 1 × 10^6^) into the anterior chambers of nude rats (*n* = 6 per group) as previously described. The minimal dose of transplanted cells that generated tumors in 50% of transplanted animals (TPD50) was estimated by non-linear regression using the logistic model to evaluate the sensitivity of tumor formation in each hiPSC line.^9^ The log_10_TPD50 of hiPSC with Matrigel was 5.256 and 5.094 cells, for 201B7 hiPSC and Ff-I01s04 hiPSC, respectively (Supplemental Fig. S1A, S1B). Although 1 × 10^6^ hiPSCs injected with Matrigel resulted in 100% teratoma formation in a short period of time, at least 1 × 10^5^ were required to form teratomas in 50% of the animals. This level of sensitivity was much lower than our expectations, so we examined whether spiking hiPSCs with human dermal fibroblasts would increase the sensitivity of teratoma formation in the anterior chamber. Fig. 3A shows the results of transplantation (*n* = 6 per group: 3 females and 3 males) with various concentrations of Ff-I01s04 iPSCs spiked with fibroblasts (total number of cells = 10^6^ each): 100% (iPSCs 1 × 10^6^), 10% (iPSCs 1 × 10^5^), 1% (iPSCs 1 × 10^4^), 0.1% (iPSCs 1 × 10^3^), 0.01% (iPSCs 1 × 10^2^), and 0% (1 × 10^6^ fibroblasts). These results are also shown in Supplemental Table S1 with the data of macroscopically and pathologically positive ratio for each concentration. A similar experiment with a second iPSC line, Ff-MH09s01, is shown in Fig. 3B. The lowest concentration for which a teratoma was detected was 1% for Ff-I01s04 and 0.1% for Ff-MH09s01, suggesting that MH-09s01 cells have a higher capability to form teratomas in a short period. We did not observe tumor formation in animals transplanted with 1 × 10^2^ iPSCs, or less, spiked with fibroblasts. The log_10_TPD50 of teratoma formation by iPSCs spiked with fibroblasts was 3.500 for Ff-I01s04 iPSCs and 2.672 for Ff-MH09s01 iPSCs (Fig. 3C, 3D). These results are also shown in Supplemental Table S2 with the data of macroscopically and pathologically positive ratio in each concentration. The log_10_TPD50 of teratoma formation by two iPSC lines (201B7, FFI01s01) without fibroblast was 3.080, while on the other hand, the log_10_TPD50 of teratoma formation by another two iPSC lines (FFI01s04, FFI-MH09s01) with fibroblasts was 4.998 (Fig. 3E, 3F). This indicates that 1 × 10^3^ iPSCs spiked in fibroblasts are sufficient to induce teratoma formation in 50% of the animals, which is two orders of magnitude more sensitive than tests without fibroblasts.

### Sensitivity of the Anterior Chamber Tumorigenesis Test using Hela Cells

**Figure 4.**
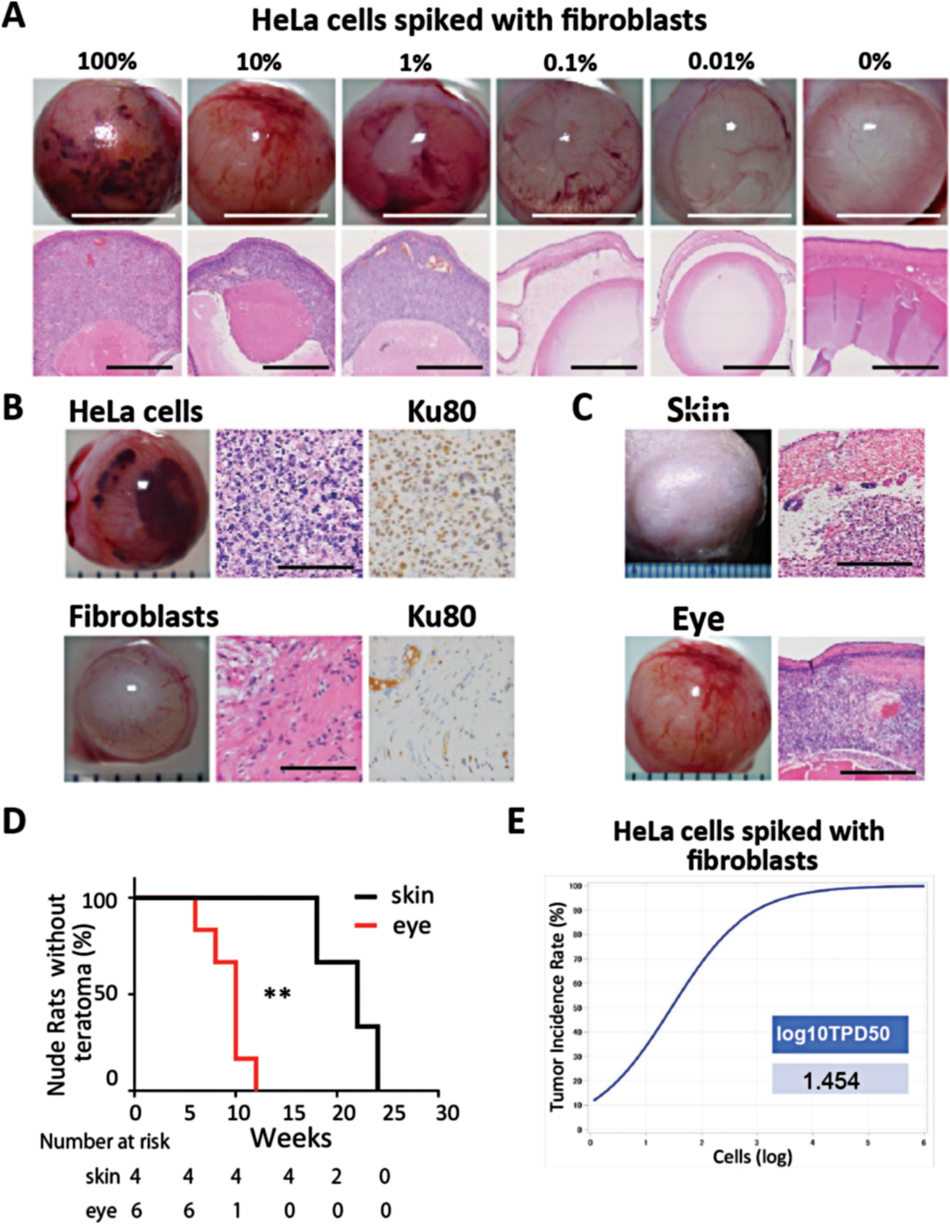
Spike tests of HeLa cells with human dermal fibroblasts. (**A**) Images of nude rat eyes transplanted with various concentrations of HeLa cells (upper panel, Scale bars = 5 mm). HE-stained section of the eyeball (lower panel) (*n* = 6). Tumors were not formed with concentrations lower than 0.01%. Scale bars = 1 mm. (**B**) Images of nude rat eyes transplanted with HeLa cells (upper panel) and with normal human dermal fibroblasts (lower panel). The HE-stained section of the eyeball confirmed the pathological characteristics of HeLa cells. Ku80 was stained to confirm the human origin of these cells. Scale bars = 50 μm. (**C**) Images of nude rats transplanted into the subcutaneous tissue (top panel) and the anterior chamber (lower panel) with HeLa cells in Matrigel. HE-stained sections confirmed the presence of HeLa cells. Scale bars = 500 μm. (D) Kaplan-Meier curve for tumor formation in nude rats injected with HeLa cells in the anterior chamber and subcutaneous tissue. The median tumor formation period in the subcutaneous area was 22 weeks (*n* = 3) (95%CI 13.74-28.92), vs. 10 in the anterior chamber (*n* = 6) (95%CI 7.16-11.6). Log-rank test (*P* = .01). (**E**) Line graph showing the minimum dose that can generate a tumor in 50% of animals transplanted with HeLa cells spiked in fibroblasts. log_10_TPD50 was 1.454.

Next, we performed the anterior chamber tumorigenicity assay using HeLa cells as a positive control for the de novo tumorigenesis assay for differentiated cells derived from iPSCs. We transplanted various concentrations of HeLa cells spiked in fibroblasts into the anterior chamber of nude rats (*n* = 6: 3 females, 3 males) and monitored animals weekly until tumor formation was observed as described above (tumor occupation in the entire anterior chamber and protrusion of the eye) (Fig. 4A). Tumors were not observed in animals transplanted with only fibroblasts. The log_10_TPD50 for HeLa was 1.454 (Fig. 4E), and 100% of the animals showed tumor formation at a concentration greater than 0.1% (1 × 10^3^ HeLa cells). Tumor formation was observed at a concentration as low as 0.01% (1 × 10^2^ HeLa cells) with an incident rate of 66.7% and an average observation period of 15.3 weeks. HE and anti-Ku80 antibody staining were performed to confirm that all tumors were of human HeLa cell origin (Fig. 4B, 4C). The high sensitivity of this test suggests that the anterior chamber tumorigenesis assay can be used for preclinical safety studies of regenerative medicine products. The results are also shown in Supplemental Table S3 with the macroscopically and pathologically positive ratio shown in each concentration.

## Discussion

The expectations for stem cell-based therapies are high, as early clinical studies have focused on unmet medical needs such as Parkinson’s disease, retinitis pigmentosa, age-related macular degeneration, and spinal cord injury.^27-31^ Many of the conditions under study have no other treatment options, and pressure to accelerate the implementation of stem cell therapy can compromise safety. Cell sources for cellular therapy include tissue stem cells and pluripotent stem cells, including embryonic stem cells (ESCs) and induced pluripotent stem cells (iPSCs). Preclinical studies of PSC-related cell therapies now require proof of the absence of residual pluripotent cells, as well as assurance that transplanted cells do not form tumors due to transformation. While more sensitive in vitro assays are available, in vivo tumorigenesis studies using the subcutaneous area is still the gold standard for clinical application.^4,5^

The in vivo tumorigenesis assay is used mainly for two purposes. One is to ensure the quality of iPSCs by showing their potential to form teratomas with three germ layers.^32^ When analyzing multiple cell lines for possible clinical use, time is of the essence. Our anterior chamber assay is easy to implement and, most importantly, allows the evaluation of teratoma formation much faster than the commonly used subcutaneous assay.^33,34^ By using the anterior chamber, teratoma formation can be observed as early as two weeks, and immunohistochemistry of enucleated eyes can be performed with ease without disrupting the tumor during the processing of pathology samples. Furthermore, our model allows semi-quantitative analysis of tumor growth over time by calculating TPD50, which is possible by macroscopic observation through the transparent cornea.

The other use of the in vivo tumorigenesis study is to examine de novo tumorigenesis due to the transformation of cells differentiated from PSCs. In this case, the observation would be much longer than in the teratoma formation study. Further studies are required to determine how long cells can persist in the anterior chamber. However, the use of extracellular matrices such as Matrigel will ensure longer retention of transplanted cells that may otherwise flow out of the anterior chamber through the trabecular meshwork.

Spiking cells with feeder cells will also enhance the sensitivity of the anterior chamber tumorigenesis model. When iPSCs were transplanted into the anterior chamber with fibroblasts, the log_10_TPD50 value was 3.26 (*n* = 12), while the value was 4.99 (*n* = 12) when hiPSCs alone were transplanted without fibroblasts. There was more than a 40-fold difference in the log_10_TPD50 values with and without fibroblasts. Similarly, there was a 23-fold increase in the log_10_TPD50 value of tumors formed by HeLa cells when spiked with fibroblasts. Although we used human fibroblasts in our study, different cells can be used according to the required objectives. De novo tumorigenesis by transformed cells is known to be affected by cellular cues from surrounding tissue. Since the anterior chamber is immune permissive, a variety of cell types can be transplanted as feeder cells without eliciting a strong immune response. For example, the anterior chamber has been used to observe the growth of ectopic tissue such as the pancreas and renal tissue.^16-20^ Therefore, de novo tumorigenesis studies for any type of PSC-derived cells could be performed by co-transplanting relevant stromal cells, eg, transplanting iPSC-derived neural tissue with glial cells. Another potential interest of transplanting in the anterior chamber is to provide an optimal environment for the in vivo differentiation for the three germ layers and, as such, could be used for promoting organoid differentiation. This application could be especially advantageous for tissues whose differentiation induction is currently challenging in 2D in vitro.

The anterior chamber has been used to study the orthoptic growth of human melanoma cells in rodents and rabbits.^35-41^ Whole-body bioluminescent imaging enables the evaluation of orthoptic tumor growth and metastasis to other organs,^42^ which can further be enhanced in combination with micro-computed tomography.^43^ Here, we show that the anterior chamber can be used for heterotopic cancer studies as well. Since the anterior chamber is immune permissive, a property termed anterior chamber immune deviation (ACAID) first reported by Streilein in mice,^44-46^ human cells can be observed for an extended period in immunodeficient animals. In addition to ACAID, the anterior chamber is also devoid of blood vessels as the cornea and lens are avascular, and the iris is covered by pigmented epithelium. Therefore, the anterior chamber is an ideal environment to observe tumor development in 3D, including vessel formation within the tumor microenvironment.

The small volume of the anterior chamber compared to the subcutaneous space may be a limitation to study certain types of tumors. We have shown that the commonly used HeLa tumor cell line can also be detected in our model with higher sensitivity than iPSC-derived teratomas. A more detailed evaluation of tumors in the anterior chamber may require additional analysis using devices such as two-photon microscopy or optical coherence tomography (OCT). However, for initial screening purposes, the transparent nature of the cornea will allow real-time observation of the tumor architecture without the need for such special imaging devices.

## Conclusion

We developed a simple, fast, and highly sensitive tumorigenesis assay protocol. This model can be used as a preclinical safety test for the formation of teratomas and de novo tumors for PSC-derived cell therapy products. Further validation of the protocol may lead to a new standardized tool for tumorigenesis studies.

## Supplementary Material

szac036_suppl_Supplementary_Table_S1

szac036_suppl_Supplementary_Table_S2

szac036_suppl_Supplementary_Table_S3

szac036_suppl_Supplementary_Figures

## Data Availability

All data related to this study are available upon request to the corresponding author.
