# Supplementary material for: The Anterior Eye Chamber as a Visible Medium for In Vivo Tumorigenicity Tests
Source: Stem Cells Transl Med. 2022 Jun 6;11(8):841–9. doi: 10.1093/stcltm/szac036 (PMC9397653; doi:10.1093/stcltm/szac036)
Supplement: szac036_suppl_Supplementary_Table_S1 [file szac036_suppl_supplementary_table_s1.docx]

**Supplemental Table 1** **Spike tests of iPSCs (FF-I01s04) with human dermal fibroblasts**

|  |  |  |  |  |  |  |
| --- | --- | --- | --- | --- | --- | --- |
| hiPSCs (Ff-I0104s) spiked with fibroblasts | | Transplanted hiPSCs ratio versus fibroblasts | | | | |
|  | | 0.01% | 0.10% | 1% | 10% | 100% |
| Macroscopically positive (%) | | 0 | 0 | 100 | 100 | 100 |
| Pathologically positive (%) | | 0 | 0 | 100 | 100 | 100 |
| Mean observation period until positivity (weeks) | | 16 | 16 | 10 | 9 | 5.5 |
|  |  |  |  |  |  |  |

hiPSCs: Human induced pluripotent stem cells
