# Supplementary material for: The Anterior Eye Chamber as a Visible Medium for In Vivo Tumorigenicity Tests
Source: Stem Cells Transl Med. 2022 Jun 6;11(8):841–9. doi: 10.1093/stcltm/szac036 (PMC9397653; doi:10.1093/stcltm/szac036)
Supplement: szac036_suppl_Supplementary_Table_S2 [file szac036_suppl_supplementary_table_s2.docx]

**Supplemental Table 2. Spike tests of hiPSCs (FF-MH09s01s) with human dermal fibroblasts**

|  |  |  |  |  |  |  |
| --- | --- | --- | --- | --- | --- | --- |
| hiPSCs (Ff-MH09s01) spiked with fibroblasts | | Transplanted hiPSCs ratio versus fibroblasts | | | | |
|  | | 0.01% | 0.10% | 1% | 10% | 100% |
| Macroscopically positive (%) | | 0 | 66.67 | 100 | 100 | 100 |
| Pathologically positive (%) | | 0 | 83.34 | 100 | 100 | 100 |
| Mean observation period until positivity (weeks) | | 16 | 12 | 10.67 | 7 | 8 |
|  |  |  |  |  |  |  |

hiPSCs: Human induced pluripotent stem cells
