# Supplementary material for: The Anterior Eye Chamber as a Visible Medium for In Vivo Tumorigenicity Tests
Source: Stem Cells Transl Med. 2022 Jun 6;11(8):841–9. doi: 10.1093/stcltm/szac036 (PMC9397653; doi:10.1093/stcltm/szac036)
Supplement: szac036_suppl_Supplementary_Table_S3 [file szac036_suppl_supplementary_table_s3.docx]

**Supplemental Table 3. Spike tests of HeLa cells with human dermal fibroblasts**

|  |  |  |  |  |  |  |
| --- | --- | --- | --- | --- | --- | --- |
| HeLa cells spiked with fibroblasts | | Transplanted HeLa cells ratio versus fibroblasts | | | | |
|  | | 0.01% | 0.10% | 1% | 10% | 100% |
| Macroscopically positive (%) | | 50 | 100 | 100 | 100 | 100 |
| Pathologically positive (%) | | 66.67 | 100 | 100 | 100 | 100 |
| Mean observation period until positivity (weeks) | | 15.33 | 13 | 11.5 | 11 | 10 |
|  |  |  |  |  |  |  |
