# Supplementary material for: The Anterior Eye Chamber as a Visible Medium for In Vivo Tumorigenicity Tests
Source: Stem Cells Transl Med. 2022 Jun 6;11(8):841–9. doi: 10.1093/stcltm/szac036 (PMC9397653; doi:10.1093/stcltm/szac036)

Figure S1*, Corresponding author: Shigeto Shimmura, TOP

A **iPSCs (201B7) with Matrigel without fibroblasts**

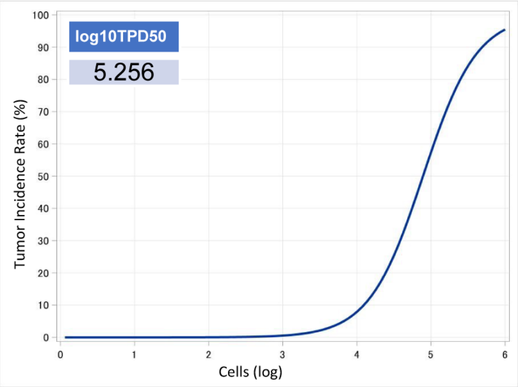

B **iPSCs (Ff-I01s01) with Matrigel without fibroblasts**

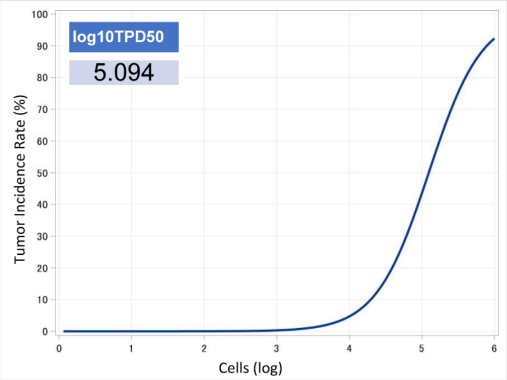

FigureS2 *, Corresponding author: Shigeto Shimmura, TOP

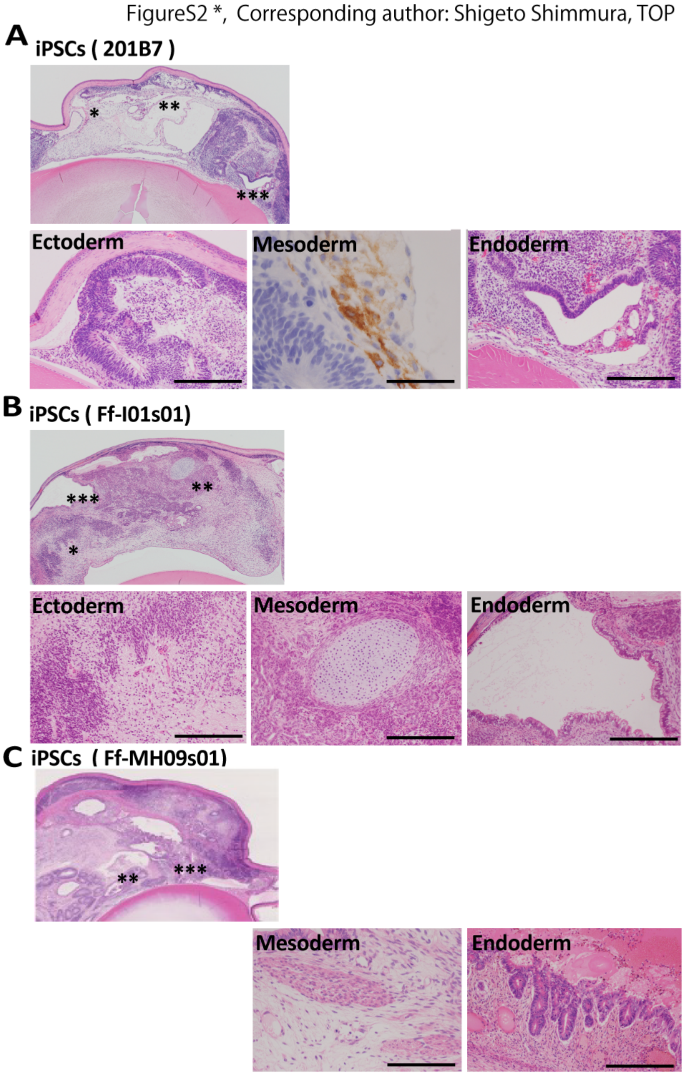

FigureS3*, Corresponding author: Shigeto Shimmura, TOP

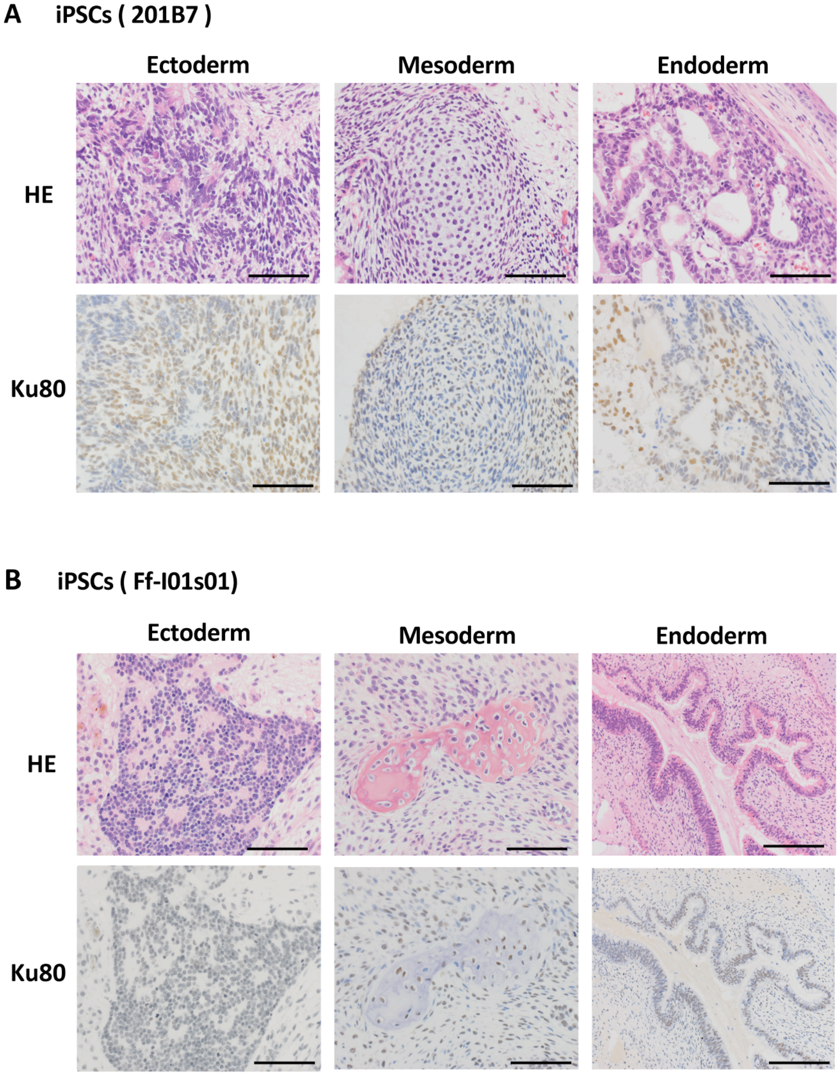

FigureS4*, Corresponding author: Shigeto Shimmura, TOP

A **iPSCs (Ff-I01s01)**

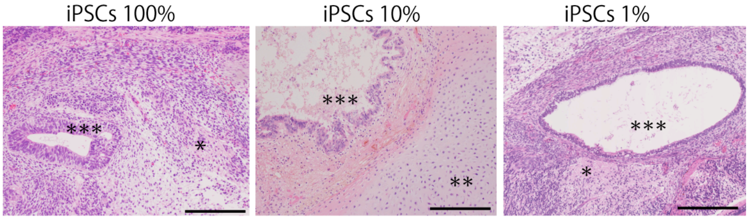

B **iPSCs (Ff-MH09s01)**

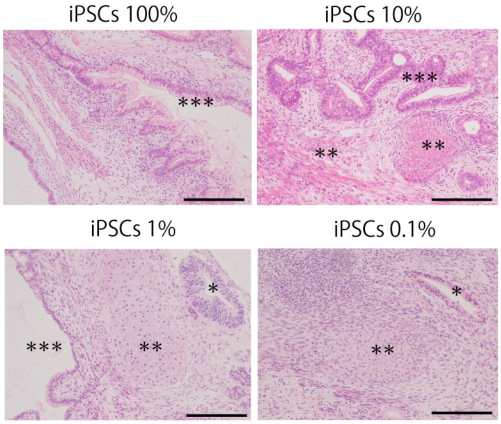

Supplement: szac036_suppl_Supplementary_Figures [file szac036_suppl_supplementary_figures.pdf]
